# Deficiency in Retinal TGFβ Signaling Aggravates Neurodegeneration by Modulating Pro-Apoptotic and MAP Kinase Pathways

**DOI:** 10.3390/ijms23052626

**Published:** 2022-02-27

**Authors:** Christina B. Bielmeier, Sabrina I. Schmitt, Nikolai Kleefeldt, Stefaniya K. Boneva, Anja Schlecht, Mario Vallon, Ernst R. Tamm, Jost Hillenkamp, Süleyman Ergün, Andreas Neueder, Barbara M. Braunger

**Affiliations:** 1 Institute of Anatomy and Cell Biology, Julius-Maximilians-University Wuerzburg, 97070 Wuerzburg, Germany; christina.bielmeier@uni-wuerzburg.de (C.B.B.); anja.schlecht@uni-wuerzburg.de (A.S.); mario.vallon@uni-wuerzburg.de (M.V.); sueleyman.erguen@uni-wuerzburg.de (S.E.); 2 Institute of Human Anatomy and Embryology, University of Regensburg, 93053 Regensburg, Germany; sabrina.schmitt@ur.de (S.I.S.); ernst.tamm@ur.de (E.R.T.); 3 Department of Ophthalmology, University Hospital Wuerzburg, 97070 Wuerzburg, Germany; kleefeldt_n@ukw.de (N.K.); hillenkamp_j@ukw.de (J.H.); 4 Eye Center, Medical Center, Faculty of Medicine, University of Freiburg, 79106 Freiburg, Germany; stefaniya.boneva@uniklinik-freiburg.de; 5 Department of Neurology, University of Ulm, 89081 Ulm, Germany; andreas.neueder@uni-ulm.de

**Keywords:** TGFβ signaling, retina, retinitis pigmentosa, neuro-/photoreceptor degeneration, MAP kinase pathway, ferroptosis

## Abstract

Transforming growth factor β (TGFβ) signaling has manifold functions such as regulation of cell growth, differentiation, migration, and apoptosis. Moreover, there is increasing evidence that it also acts in a neuroprotective manner. We recently showed that TGFβ receptor type 2 (*Tgfbr2*) is upregulated in retinal neurons and Müller cells during retinal degeneration. In this study we investigated if this upregulation of TGFβ signaling would have functional consequences in protecting retinal neurons. To this end, we analyzed the impact of TGFβ signaling on photoreceptor viability using mice with cell type-specific deletion of *Tgfbr2* in retinal neurons and Müller cells (*Tgfbr2^ΔOC^*) in combination with a genetic model of photoreceptor degeneration (VPP). We examined retinal morphology and the degree of photoreceptor degeneration, as well as alterations of the retinal transcriptome. In summary, retinal morphology was not altered due to TGFβ signaling deficiency. In contrast, VPP-induced photoreceptor degeneration was drastically exacerbated in double mutant mice (*Tgfbr2^ΔOC^*; VPP) by induction of pro-apoptotic genes and dysregulation of the MAP kinase pathway. Therefore, TGFβ signaling in retinal neurons and Müller cells exhibits a neuroprotective effect and might pose promising therapeutic options to attenuate photoreceptor degeneration in humans.

## 1. Introduction

Retinal degeneration is among the leading causes of blindness worldwide [1,2]. Intriguingly, a multitude of different causative pathomechanisms such as various genetic mutations in patients suffering from retinitis pigmentosa [3,4] or as a consequence of age-related macular degeneration (AMD) [5,6] and systemic diseases like diabetes [7], promote degeneration of retinal neurons such as the rod and cone photoreceptor cells. Photoreceptor cells are the light-sensitive cells of the retina and as such are responsible for visual perception [8]. Morphologically, these cells exhibit an outer segment that is connected through a cilium with the inner segment, a perikaryon which is located in the outer nuclear layer (ONL) and a synaptic ending located in the outer plexiform layer (OPL) of the retina [8,9]. Photoreceptor degeneration typically results in a thinning of the ONL concomitant with the loss of the inner and outer segments, resulting in a loss of visual function up to complete blindness [2,6]. Due to the multiple pathomechanisms of photoreceptor degeneration, it is still challenging to understand and intervene in the molecular mechanisms leading to their degeneration, with the overall goal of mitigating it. We recently analyzed the retinal transcriptome of the VPP mouse model, a genetic mouse model carrying a transgenic rhodopsin V20G/P23H/P27L (VPP), which results in photoreceptor degeneration as observed in autosomal dominant retinitis pigmentosa [9,10]. In the course of that study we aimed to identify molecular key factors and signaling pathways that predominantly influence the course of photoreceptor degeneration [9]. Amongst other findings, our data indicated a clustering of significantly dysregulated genes coding for components of potentially neuroprotective pathways such as the transforming growth factor β (TGFβ) signaling pathway [9]. Moreover, we particularly identified a significant upregulation of TGFβ receptor type 2 (TGFBR2) during photoreceptor degeneration [9].

TGFβ signaling controls a plethora of cellular responses such as proliferation, differentiation, tissue homeostasis, morphogenesis and regeneration [11]. In addition, there is increasing evidence that it also has neuroprotective properties [12,13,14,15,16,17]. TGFβ signaling is initiated by binding of specific ligands (e.g., TGFβ1–3) to the type II transmembrane receptor (TGFBR2), a serine/threonine kinase that builds a heterodimer with the further signaling of the type I receptor (TGFBR1) [18]. Upon activation, TGFBR2 phosphorylates the TGFBR1 kinase domain resulting in phosphorylation of its intracellular downstream effectors SMAD2 and SMAD3 [19]. Subsequently, SMAD2/SMAD3 form a complex with SMAD4 and translocate into the nucleus to promote the activation of the canonical TGFβ signaling pathway [20] through transcriptional regulation of TGFβ-dependent target genes [19]. In contrast, non-canonical TGFβ signaling pathways involves the activation of various branches of MAP kinase (MAPK) pathways, Rho-like GTPase signaling pathways, and phosphatidylinositol-3-kinase (PI3K)/AKT pathways that regulate target genes [20,21].

We have previously identified retinal neurons and Müller cells as the cell populations in which *Tgfbr2* was notably upregulated following photoreceptor degeneration [9]. Therefore, in this study we conditionally deleted TGFβ signaling specifically in retinal neurons and Müller cells. To induce photoreceptor degeneration, we used the VPP mouse model [9,10] and asked the question whether additional deletion of TGFβ signaling in this model might result in a higher susceptibility of photoreceptors to VPP-induced degeneration and what the changes on the retinal transcriptome would be. In summary, we provide evidence for an important role of TGFβ signaling for photoreceptor survival. Deletion of TGFβ signaling in retinal neurons and Müller cells sensitizes the retinal neurons to degeneration, potentially through ferroptosis, and enhances neurodegeneration by shifting the MAPK signaling pathway towards its pro-apoptotic side.

## 2. Results

### 2.1. Deletion of TGFβ Signaling in Retinal Neurons and Müller Cells in Health and Disease

**Figure 1:**
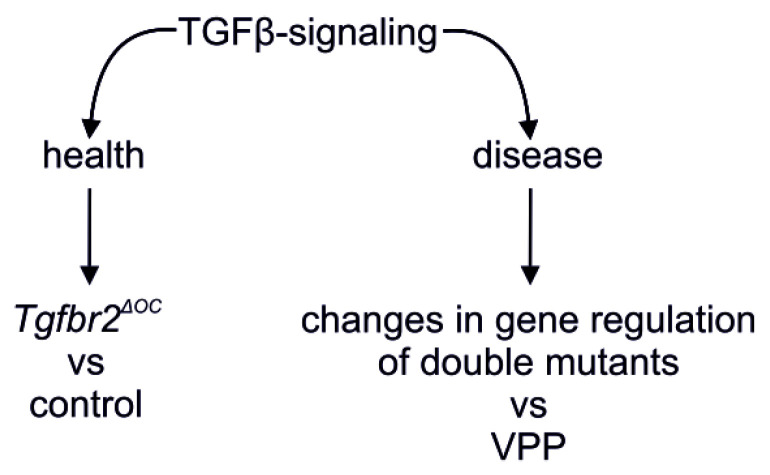
Schematic depicting the two experimental conditions that we addressed in this study. The role of TGFβ signaling in the adult, healthy retina (**left**) and its role in disease, such as VPP-induced photoreceptor degeneration (**right**).

In this study, we investigated the impact of TGFβ signaling on the retina in healthy and neurodegenerative retinae with the overall aim of identifying TGFβ-dependent molecular key factors promoting neuroprotection (Figure 1). *Tgfbr2^ΔOC^* and *Tgfbr2^fl/fl^* mice (see material and methods) were crossbred with hemizygous VPP mice to obtain double mutant mice with a genetically induced photoreceptor degeneration [10]. The resulting offspring analyzed in this study were as follows: Control mice (expressing wildtype rhodopsin and carrying *Tgfbr2^fl/fl^* alleles); *Tgfbr2^ΔOC^* mice (expressing wildtype rhodopsin and harboring a Cre-mediated deletion of *Tgfbr2* in retinal neurons and Müller cells); *Tgfbr2^fl/fl^*;VPP mice (henceforth termed ‘VPP mice’; expressing the VPP rhodopsin mutant protein) and *Tgfbr2^ΔOC^*; VPP mice (henceforth termed ‘double mutant mice’; expressing the VPP transgene in combination with Cre-mediated deletion of *Tgfbr2* in retinal neurons and Müller cells).

*Tgfbr2^ΔOC^* mice allowed us to analyze the effects of deletion of TGFβ signaling in otherwise healthy retinae by analyzing the morphology and transcriptomic changes of *Tgfbr2^ΔOC^* mice in comparison to control mice (Figure 1, left side). In addition, we analyzed the effects of the deletion of TGFβ signaling (*Tgfbr2^ΔOC^*) in conjunction with a model of retinal degeneration (VPP). To this end, we compared morphological and molecular changes in the double mutants, in which TGFβ signaling was additionally deleted, to those of the VPP mice (Figure 1, right side).

### 2.2. Deletion of TGFβ Signaling in Healthy Retinae Is Not Sufficient to Cause Morphological Changes

First, we studied the retinal morphology of *Tgfbr2^ΔOC^* and control animals to investigate whether the deletion of *Tgfbr2* might have an impact on it. The verification of the successful deletion of TGFBR2 and its downstream effector pSMAD3 in *Tgfbr2^ΔOC^* retinae has already been published in [12]. In the current project, we additionally performed *Tgfbr2* in situ hybridization on retinal sections and show that *Tgfbr2* signals were dramatically reduced in *Tgfbr2^ΔOC^* retinae (Figure S1).

**Figure 2:**
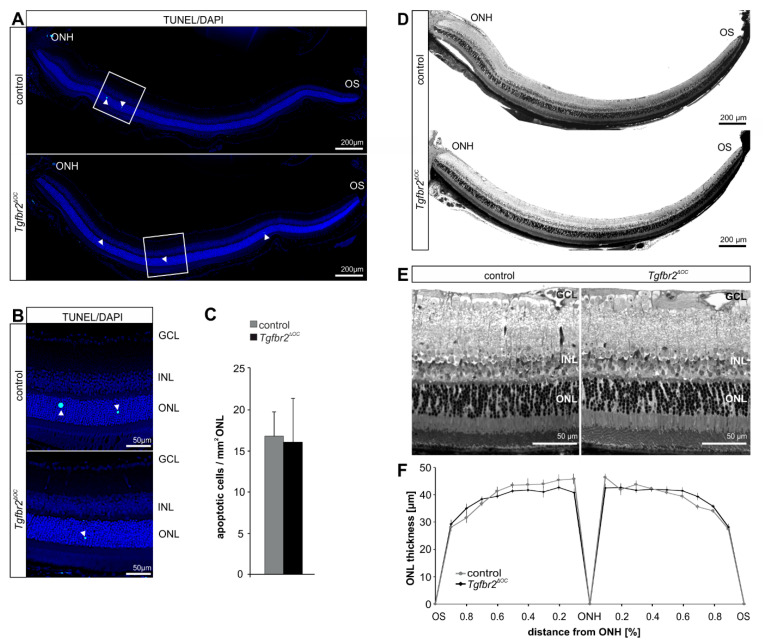
Cell death, retinal morphology and morphometry in *Tgfbr2^ΔOC^* mice. (**A**,**B**) Mid-horizontal sections of TdT-mediated dUTP-biotin nick end (TUNEL)-labeled (green, arrowheads) retinae of one-month-old control and *Tgfbr2^ΔOC^* animals (**A**). Detailed magnification (**B**) of the boxed areas in (**A**). Cell nuclei were stained with DAPI (blue). (**C**). Total number of TUNEL-positive cells normalized to mm^2^ ONL. Controls: *n* = 9; *Tgfbr2^ΔOC^*: *n* = 9. (**D**)**.** Richardson-stained, mid-horizontal semithin sections of the posterior eye segment of three-month-old control and *Tgfbr2^ΔOC^* mice. (**E**,**F**)**.** The detailed magnification of the central retina (**E**) shows a regular morphology of control and *Tgfbr2^ΔOC^* animals. The thickness of the ONL was measured on mid-horizontal semithin sections at defined measure points and the mean values were plotted in the spider-diagram in (**F**). Controls *n* = 6; *Tgfbr2^ΔOC^*: *n* = 13. GCL = ganglion cell layer; INL = inner nuclear layer; ONL = outer nuclear layer; ONH = optic nerve head; OS = ora serrata. Data are means ± SEM. No significant changes were detected (Student’s *t*-test).

When analyzing the number of degenerating, TUNEL-positive photoreceptor cells in the outer nuclear layer (ONL) of one-month-old mice, we did not find significant differences in their number (controls: 16.69 ± 2.94, *n* = 9; *Tgfbr2^ΔOC^*: 15.98 ± 5.29, *n* = 9, *p* = 0.9) (Figure 2A–C). Moreover, the morphology of retinae from three-month-old mice did not show obvious morphological alterations between control and *Tgfbr2^ΔOC^* animals. Morphometric analyses of the thickness of ONL revealed a largely comparable ONL thickness between control and *Tgfbr2^ΔOC^* mice (Figure 2D–F).

### 2.3. Deletion of TGFβ Signaling in Healthy Retinae Is Not Sufficient to Induce Major Transcriptional Changes

To investigate the impact of TGFβ signaling on the retinal transcriptome in healthy retinae (Figure 1, left side), we performed RNA sequencing (RNAseq) analyses of control (*Tgfbr2^fl/fl^*) and *Tgfbr2^ΔOC^* retinae. Out of the total of 54,532 genes in the Ensembl gene annotation for mouse (Mus musculus GRCm38 v. 94) we found 30,796 genes to be expressed in the retina.

**Figure 3:**
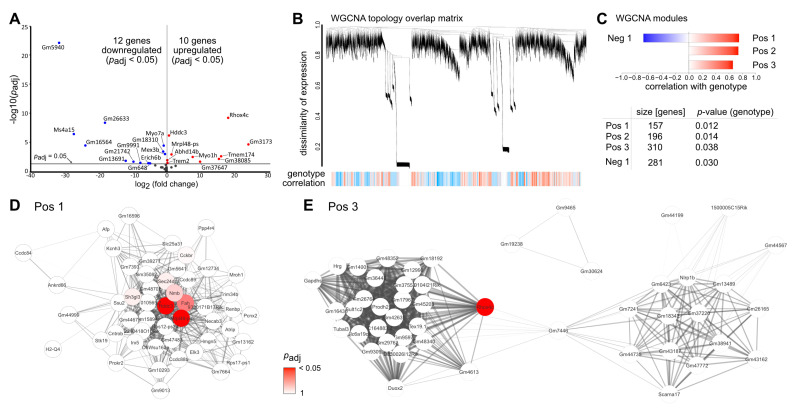
Transcriptome analysis: TGFβ effects in the adult, healthy retina. (**A**). RNAseq analysis identified 12 significantly down- and 10 significantly up-regulated genes, respectively, in retinae of three-month-old *Tgfbr2^ΔOC^* animals (Benjamini-Hochberg adjusted *p*-values; *p_adj_*). (**B**). WGCNA analysis of *Tgfbr2^ΔOC^* and control animals. Blue color in the lower panel (genotype correlation) indicates lower expression and red color indicates higher expression in *Tgfbr2^ΔOC^* mice. (**C**). Three positively correlated modules (indicating higher expression in *Tgfbr2^ΔOC^* animals) and one negatively correlated module (indicating lower expression in *Tgfbr2^ΔOC^* animals) were identified to be significantly associated with the genotype. (**D**,**E**) Intra-module analysis of the Pos1 (**D**) and Pos3 (**E**) modules. The 50 highest connected (intramodular connectivity) genes with the 500 strongest connectivities per module are shown. Red colored genes were shown to be significantly upregulated in the DESeq2 analysis. The intensity of the fill color is inversely related to the adjusted *p*-value in the DESeq2 analyses.

Only 22 genes were differentially expressed in *Tgfbr2^ΔOC^* retinae compared to control animals (12 down- and 10 upregulated, Figure 3A, cut off criteria: Benjamin-Hochberg adjusted *p*-value (*p_adj_*) < 0.05, Table S1). Amongst others, Myosin VIIA (*Myo7a*) which is a member of the myosin gene family and associated with the mouse shaker-1 phenotype and the human Usher syndrome 1B [22] was significantly downregulated in *Tgfbr2^ΔOC^* retinae. Moreover, HD Domain Containing 3 (*Hddc3*) and Triggering Receptor Expressed on Myeloid cells 2 (*Trem2*) were significantly upregulated in *Tgfbr2^ΔOC^* retinae. *Hddc3* (also known as *Mesh1*) is expressed in a broad range of cells in the body (www.proteinatlas.org, accessed on 1 December 2021) with attributed functions e.g., in body growth, resistance to starvation and ferroptosis [23,24]. *Trem2* constitutes an innate immune receptor, preferentially expressed by microglia, and involved in inflammation and microglial-mediated phagocytosis of e.g., apoptotic neurons [25].

Moreover, we did not detect significant alterations in the expression of Müller glia cell specific markers such as Glutamine Synthetase (*Glul*), Integrin beta-1 (*Itgb1*, also known as *Cd29*) or Retinaldehyde-binding Protein 1 (*Rlbp1*, also known as cellular Retinaldehyde-binding Protein (*Cralbp*)) in *Tgfbr2^ΔOC^* animals compared to controls (Table S1), indicating that cellular maintenance and homeostasis of Müller glia cells was not affected by the deletion of TGFβ signaling.

As gene ontology analyses and pathway enrichment analyses do not work reliably for small sets of genes, we performed weighted gene correlation network analysis (WGCNA) to potentially identify more subtle genotype-specific patterns of dysregulation, potential upstream regulators and involved signaling pathways in *Tgfbr2^ΔOC^* retinae.

WGCNA identifies co-regulated genes by clustering them into modules based on their similarity of expression. This approach is able to uncover more subtle changes and patterns as it does not rely on the traditional dysregulation analysis and the problem of correction for multiple comparisons. Additionally, the network analysis allows the identification of biological key players, e.g., regulatory proteins driving a certain pathway.

The topology overlay matrix, demonstrating the co-regulation of gene expression for *Tgfbr2^ΔOC^* and control animals, as well as the identified modules (clusters of co-regulated genes) are shown in Figure 3B,C. The analysis identified four significantly associated modules (three positively correlated with the genotype, i.e., higher expression in *Tgfbr2^ΔOC^* retina (Pos1, 2, 3) and one negatively correlated, i.e., lower expression in *Tgfbr2^ΔOC^* retina (Neg1) (Figure 3D,E and Figure S2A,B).

The Pos1 module contained 157 genes, and amongst those, the HD Domain Containing 3 (*Hddc3*) and mitochondrial ribosomal protein L48 pseudogene (*Mrpl48ps*) were central hub genes in WGCNA analyses and significantly dysregulated in Deseq2 analyses (Figure 3D (dysregulated genes are highlighted in red) and Tables S1 and S2). In the Pos2 module, we found a clustering of 196 genes and in the Neg1 module a clustering of 310 genes, (Figure S2A,B and Tables S1 and S2). However, none of them were dysregulated in DEseq2 analyses (Figure S2A,B and Tables S1 and S2). The Pos3 module contained 281 genes with Reproductive Homeobox 4C (*Rhox4c*) as the only significantly upregulated gene in this module Figure 3E and Tables S1 and S2).

In summary, WGCNA analyses did not detect a significant enrichment of genes coding for certain biological processes or pathways, which is consistent with our dysregulation analysis (DESeq2). We therefore conclude that deletion of TGFβ signaling in retinal neurons and Müller cells in the adult and healthy retina affects the retinal transcriptome only very mildly.

### 2.4. Deletion of TGFβ Signaling Increases the Susceptibility of Photoreceptors to Vpp-Induced Neurodegeneration

**Figure 4:**
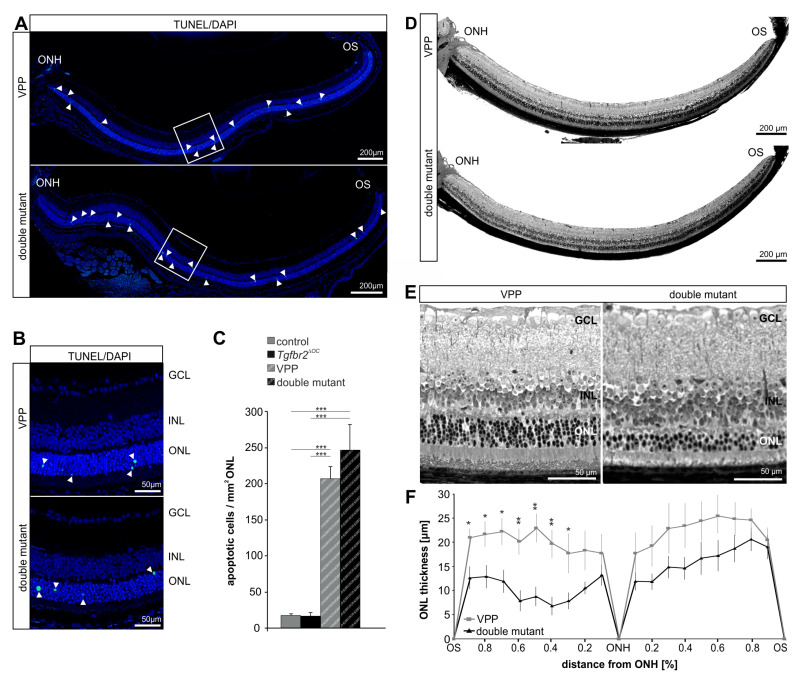
TGFβ signaling affects cell death, retinal morphology and morphometry in neurodegenerative retinae. (**A**). Mid-horizontal sections of one-month-old TdT-mediated dUTP-biotin nick end (TUNEL)-labeled (green, arrowheads) retinae (**A**) and detailed magnification (**B**) of the boxed areas in (**A**). Cell nuclei were stained with DAPI (blue). (**C**). Total number of TUNEL-positive cells normalized to mm^2^ ONL. VPP *n* = 10; double mutant *n* = 7. Data are means ± SEM. ANOVA with Bonferroni *post-hoc* analysis; *** *p* < 0.001. (**D**). Richardson-stained, mid-horizontal semithin sections of three-month-old VPP and double mutant mice. (**E**). The detailed magnification of the central retina shows the thinner ONL in the double mutant animal compared to the ONL of the VPP animal. (**F**). The thickness of the ONL was measured on mid-horizontal semithin sections from VPP and double mutant retinae at defined measure points and the mean values were plotted in the spider-diagram. Controls *n* = 9; VPP *n* = 6. Data are means ± SEM. student’s *t*-test. * *p* ≤ 0.05, ** *p* ≤ 0.01. GCL = ganglion cell layer; INL = inner nuclear layer; ONL = outer nuclear layer; ONH = optic nerve head; OS = ora serrata.

Next, we investigated whether deletion of TGFβ signaling in retinal neurons and Müller cells might impact the susceptibility of photoreceptors to VPP-induced degeneration. When analyzing the number of degenerating photoreceptor cells in the outer nuclear layer (ONL) of one-month-old VPP and double mutant mice, both groups demonstrated significantly more TUNEL-positive cells in the ONL (VPP mice: 205.76 ± 16.89, *n* = 10; double mutant mice: 245.61 ± 35.59, *n* = 7) compared to control (16.69 ± 2.94, *n* = 9, *p* < 0.001) and compared to *Tgfbr2^ΔOC^* retinae (15.98 ± 5.29, *n* = 9, *p* < 0.001). Yet, double mutant mice (Figure 4A–C) demonstrated a slightly higher number of degenerating photoreceptors compared to VPP mice, although this alteration did not reach significance. Next, we analyzed whether the observed transcriptional alterations and the slight increase in degenerating photoreceptor cells might impact the retinal morphology of three-month-old double mutant animals. As expected, mice carrying the VPP transgene showed a significant thinning of the ONL (Figure 4D,E) compared to controls (*p* < 0.02) and *Tgfbr2^ΔOC^* mice (*p* < 0.001), confirming the expected VPP-induced degeneration of photoreceptors [9,10]. Intriguingly, double mutant retinae demonstrated a significantly thinner ONL compared to VPP retinae (*p* < 0.03) (Figure 4D–F), showing that deletion of TGFβ signaling in retinal neurons and Müller cells exacerbates VPP-induced photoreceptor degeneration.

### 2.5. TGFβ-Mediated Effects on Vpp-Induced Transcriptomic Alterations

**Figure 5:**
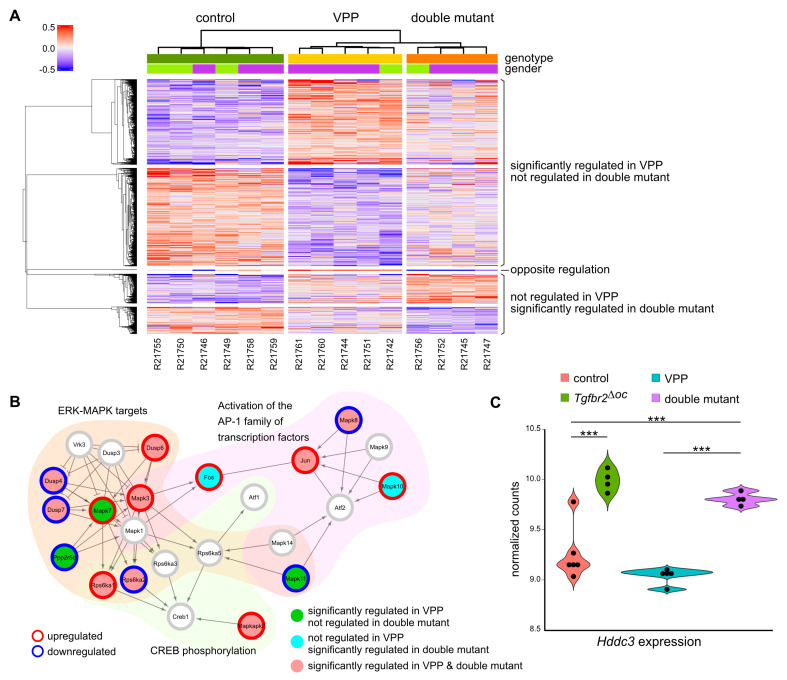
Transcriptome analysis: TGFβ effects in VPP-induced neurodegeneration. (**A**). Heatmap showing the deviation of expression from the mean of all significantly dysregulated genes in any of the pairwise comparisons: VPP and double mutant, respectively, against control retinae. Significantly dysregulated genes, which were significantly dysregulated (up- or down-regulated) in the same way in both comparisons are not shown here, but can be found in Figure S3. R21742-R21761 = RNAseq sample number. (**B**). Visualization of the Reactome MAPK targets/ Nuclear events mediated by MAP kinases signaling pathway. We converted the Reactome pathway into functional interaction networks. For each network, genes were colored according to the comparison (see legend) and their dysregulation state (white—not significantly dysregulated; red—significantly upregulated; and blue—significantly downregulated). (**C**). Violin blots with individual data points showing *Hddc3* gene expression data from the RNAseq as normalized counts for control, *Tgfbr2^ΔOC^*, *VPP* and double mutant animals. ANOVA with Bonferroni *post-hoc* analysis; *** *p* < 0.001.

We have previously shown that VPP mice display huge alterations of the retinal transcriptome with thousands of significantly dysregulated genes [9]. To investigate TGFβ-mediated effects during VPP-induced photoreceptor degeneration (as illustrated in Figure 1, right side), we analyzed changes in the regulation patterns of gene expression in the retinae of double mutant animals (VPP plus TGFβ signaling deletion) in comparison to the gene regulation patterns in VPP mice (Figure 5A). To this end, we clustered significantly dysregulated genes (Benjamin-Hochberg adjusted *p*-value *p_adj_* < 0.05) of any of the two genotype analyses (double mutant vs. controls mice and VPP vs. control mice) into genotype/regulation specific groups. We found that 647 genes were regulated in the double mutant mice, but not in the VPP mice (Table 1). In contrast, 2106 genes were regulated in the VPP mice, but not in the double mutant mice (Table 2). While deletion of TGFβ signaling led to the aforementioned changes in the gene regulation patterns, the majority, namely 7148 significantly dysregulated genes, were similarly regulated in both double mutant and VPP mice (Figure S3).

The analysis of the double mutant specific gene regulation patterns can be found in Table 1 and Table S3: ‘VPPnot_doubleDown’ and ‘VPPnot_doubleUp’. Gene ontology and pathway enrichment analyses showed that, amongst other findings, an activation of the activator protein 1 (AP-1) family of transcription factors’ in double mutants (Table 1). This pathway is part of the Reactome pathway ‘mitogen-activated protein kinase (MAPK) targets Nuclear events mediated by MAP kinases’ (Figure 5B). We found significant upregulation of the neuronal specific *Mapk10* (also known as c-Jun N-terminal kinase 3 (*JNK3*)) and *Fos* (Fos proto-oncogene, also known as AP-1 transcription factor subunit) in double mutants (Figure 5B, turquoise dots). Moreover, the ‘interleukin (IL) -6 signaling pathway’ was amongst the top hits in the pathway enrichment analyses (Table 1). IL6 is a pleotropic cytokine and is involved in a multitude of central nervous system (CNS) pathologies including injury and neurodegeneration [26]. In addition, ‘negative regulation of synapse organization’ was also amongst the top hits in the pathway enrichment analyses (Table 1), potentially indicating a reduced number of existing synapses in double mutant retinae as a result of increased neurodegeneration.

In contrast, 2106 genes were significantly dysregulated in VPP mice, but not in double mutant mice (Table 2 and Table S3: ‘VPPdown_doubleNot’ and ‘VPPup_doubleNot’). In these clusters, gene ontology enrichment analyses indicated, amongst others, downregulation of genes controlling e.g., processes in mRNA processing and biology and an upregulation of genes that clustered e.g., for negative regulation of T cell migration, and cell cycle negative regulation by p75 neurotrophin receptor (Table 2). In addition, we found a significant downregulation of *Mapk11* (Figure 5B, green dots), which is one of the p38 MAPKs [27], while *Mapk7* (Figure 5B, green dots), which is a component of the ERK signaling pathway and associated with AP1 signaling [28], was significantly upregulated in VPP mice but not in double mutants.

Interestingly, we identified only two genes that were oppositely regulated in double mutant and VPP mice: Mitochondrial Ribosomal Protein L48 Pseudogene (*Mrpl48-ps*; log_2_ fold changes: VPP: −0.60, double mutant: 1.38) and Myosin VIIa (*Myo7a*; log_2_ fold changes: VPP: 0.41, double mutant: −0.41) (Table S3). *Myo7a* is amongst others critical for renewal of the outer photoreceptor disks, distribution and migration of RPE melanosomes and phagosomes [29]. As mentioned above, mutations in *Myo7a* are associated with the Usher syndrome I, a genetically heterogeneous condition that is characterized by congenital sensorineural deafness, absent vestibular function and prepubertal onset of progressive retinitis pigmentosa leading to blindness [30].

Moreover, *Hddc3*, which was significantly higher expressed in *Tgfbr2^ΔOC^* retinae (see above), was also significantly higher expressed in double mutant retinae compared to control, or VPP only mutant retinae (Figure 5C).

## 3. Discussion

The data of this study show that the deficiency of TGFβ signaling in retinal neurons and Müller cells in adult, healthy mice affects the retinal transcriptome only very mildly and does not result in obvious morphological alterations in the post-developmental retina. However, during VPP-induced photoreceptor degeneration, upregulation of genes involved in neurodegeneration and downregulation of genes essential for cellular maintenance and homeostasis were exacerbated by the additional deletion of TGFβ signaling. These effects culminate in enhanced vulnerability and degeneration of photoreceptors, resulting in a significantly thinner ONL.

### 3.1. TGFβ Signaling in Retinal Development and in the Healthy, Adult Retina

TGFβ signaling has a plethora of different functions such as cell-cycle control, cell differentiation, and regulation of early development [31,32,33,34]. As the Cre recombinase in *Tgfbr2^ΔOC^* retinae is constitutively expressed from embryonic day 10.5 in all cells deriving from the inner layer of the optic cup e.g., retinal neurons and Müller cells [35], we addressed potential developmental-related aspects in the *Tgfbr2^ΔOC^* model in one of our previously published manuscripts [12]. We showed that *Tgfbr2^ΔOC^* animals exhibited a higher degree of degenerating neurons particularly affecting the inner retinal neurons (retinal ganglion cells and neurons of the INL) during developmental programmed cell death of the retina [12]. Consequently, adult *Tgfbr2^ΔOC^* retinae harbor mild, developmental-related alterations, such as a reduced number of retinal ganglion cells or neurons of the INL [12]. However, only a negligible percentage of photoreceptors undergo programmed cell death during retinal development [36]. Accordingly, thicknesses of the ONL between control and *Tgfbr2^ΔOC^* of two-month-old animals [12] and three-month-old animals (data of this publication, Figure 3F) were largely comparable.

Given the manifold properties of TGFβ signaling in cellular homeostasis, it was surprising to detect only 22 dysregulated genes in the RNAseq analyses of healthy, adult *Tgfbr2^ΔOC^* animals when compared to control mice. This finding clearly indicates that in the healthy, post-developmental retina, TGFβ signaling is not essential for cellular maintenance and homeostasis of retinal neurons and Müller cells. However, our transcriptome analyses were performed using total retinal tissue, a mixed tissue containing cell types such as microglial cells, endothelial cells, perivascular cells, and astrocytes, which were not affected by the deletion of TGFβ signaling in *Tgfbr2^ΔOC^* animals. Therefore, subtle transcriptional changes affecting e.g., only Müller cells or a subpopulation of retinal neurons might not have been detected by our approach. Still, we found genes like *Myo7a* (Myosin VIIA), which is associated with Usher syndrome [22], to be dysregulated in *Tgfbr2^ΔOC^* retinae. Thus, it is reasonable to speculate that dysregulation of TGFβ signaling might have an impact on the cellular ‘buffer capacity’ against cytotoxic insults potentially aggravating the course of human diseases such as Usher syndrome, as well.

### 3.2. TGFβ Signaling Mediated Effects in Retinal Neurodegeneration

We described the molecular effects of VPP-induced photoreceptor degeneration on the retinal transcriptome using RNAseq analyses in our previously published manuscript [9]. In this study, the deletion of TGFβ signaling in VPP-induced photoreceptor degeneration resulted in a dysregulation of more than 600 genes in double mutant retinae, which were not differentially expressed in the VPP retinae alone. Gene ontology analyses showed, amongst others, an upregulation of the AP-1 family of transcription factors associated signaling in double mutant retinae. AP-1 transcription factor is associated with a broad range of apoptosis-related interactions [37]. In particular, in our data *Mapk11* was significantly downregulated in VPP retinae but not in double mutants. *Mapk11* is one of the p38 MAPKs and plays an important role in cellular responses to, for example, proinflammatory cytokines or physical stress [27], and in the regulation of Tumor necrosis factor (TNF) expression in monocytic cells [38].

Moreover, *Mapk10* and *Fos* were significantly upregulated in double mutant retinae (Figure 5B). *Mapk10* plays a regulatory role during neuronal apoptosis [39], and the transcription factor *Fos*, which is part of the AP-1 transcription factor complex, and as such orchestrates expression of target genes that e.g., regulate neuronal cell death versus survival [40,41].

In contrast, *Mapk7*, which is a component of the ERK signaling pathway and associated with AP-1 signaling (Figure 5B) [28], was significantly upregulated in VPP retinae but not in double mutants. *Mapk7* (also known as ERK5) regulates gene expression upon activation in response to various growth factors such as the neurotrophins nerve growth factor (NGF) and brain derived neuroprotective factor (BNDF), or in response to oxidative stress, finally contributing to anti-apoptotic signaling [28]. Thus our data indicate that a deficiency of TGFβ signaling in retinal neurons and Müller cells results in an imbalance of MAPK associated signaling pathways, finally shifting its impact towards the pro-apoptotic side. It is reasonable to assume that this effect is directly related to TGFβ signaling, as particularly non-canonical TGFβ signaling regulates the transcription of target genes, amongst others, through activation of MAPK pathways [20].

Moreover, gene ontology analyses suggested an upregulation of ‘cell cycle negative regulation of p75 neurotrophin receptor’ in VPP, but not in double mutant retinae. P75 neurotrophin receptor is one of the neurotrophin receptors, mediating predominately pro-apoptotic effects [34,42]. We have recently demonstrated that expression of the neurotrophin *Ngf* is dependent upon TGFβ2 treatment in vitro and is significantly enhanced in the juvenile retina of a mouse model with increased TGFβ signaling activity [12]. Hence, the fact that ‘p75 neurotrophin receptor’ was amongst the genes that were upregulated in VPP but not regulated in double mutant retinae might point towards an interaction of neurotrophin and TGFβ signaling, as postulated in previously published manuscripts from our group [12,34] and others [43,44,45].

*Hddc3* was more highly expressed in *Tgfbr2^ΔOC^*. This effect persisted in the comparison of double mutant and VPP retinae, where *Hddc3* expression was also induced in the double mutants due to the deletion of TGFβ signaling. Published data show that overexpression of HDDC3 (also known as MESH1) sensitize cells to ferroptosis [24]. Another study links ferroptosis to neuronal cell death [46]. Taken together, deficiency of TGFβ signaling in retinal neurons and Müller cells might sensitize the retina towards ferroptosis associated neuronal cell death. Accordingly, our data clearly demonstrate that TGFβ signaling in retinal neurons and Müller cells contributes in a neuroprotective manner on photoreceptor survival in the adult retina. Moreover, we recently showed that TGFβ2 treatment of in vitro cultures of retinal neurons improved their survival significantly, an effect that could be reversed to that of untreated controls, when SIS3, an inhibitor of SMAD3 phosphorylation, was added [12]. Other groups showed that adeno-associated virus (AAV)-mediated delivery of TGFβ1 rescued degenerating cone photoreceptor cells in mouse models mimicking retinitis pigmentosa [47], and our previously published manuscript demonstrated that TGFβ signaling protected inner retinal neurons from ontogenetic cell death during retinal development [12].

The question remains whether the observed neuroprotective effect was mediated directly (TGFβ signaling in photoreceptors) and/or indirectly (TGFβ signaling in Müller cells and non-photoreceptor retinal neurons). In this context, we recently showed that primary retinal neurons, isolated from newborn pups and treated with TGFβ2, demonstrated significantly higher survival in vitro [12], indicating that TGFβ signaling regulates the survival of retinal neurons directly. However, TGFβ signaling in Müller cells and/or non-photoreceptor retinal neurons may induce the release of paracrine neuroprotective factors mediating the observed effects on photoreceptors. Therefore, future studies using cell type-specific knockout mouse models of TGFβ signaling are needed to answer this question.

## 4. Conclusions

In this study, we showed that the deletion of TGFβ signaling in retinal neurons and Müller cells affects the retinal transcriptome of adult, healthy mice in only a very minor way, without obvious morphological alterations of the post-developmental retina. In contrast, the concurrent expression of mutant rhodopsin (VPP) [10] and deletion of TGFβ signaling resulted in a significantly thinner ONL. The predominant changes in the regulation of gene expression in these mice indicate the dysregulation of cellular homeostasis and the upregulation of pathways involved in neurodegeneration. Moreover, gene ontology analyses found that TGFβ signaling deficiency mediates a shift in the expression of MAPK signaling pathway regulators from pro-survival to pro-apoptosis. Conversely, the stimulation of TGFβ signaling or activation of pro-survival MAPK signaling pathways in retinal neurons or in Müller cells might be promising approaches to attenuate the degeneration of photoreceptors in diseases such as retinitis pigmentosa or age-related macular degeneration.

## 5. Material and Methods

### 5.1. Mice

All procedures conformed to the tenets of the National Institutes of Health Guidelines on the Care and Use of Animals in Research, the EU Directive, 2010/63/E and institutional guidelines. The mice were on a 129 SV background and kept in a 12 h light/dark cycle. Mice carrying two floxed *Tgfbr2* alleles (*Tgfbr2^fl/fl^*) [48] were crossbred with α-Cre; *Tgfbr2^fl/fl^* mice [12,35] hemizygous for the α-Cre transgene. The α-Cre transgene contains a Cre recombinase under control of the retina-specific α enhancer and minimal promoter of the Pax6 gene [35]. The resulting α-Cre, *Tgfbr2^fl/fl^* mice (for simplicity referred as *Tgfbr2^ΔOC^*) had recombined and inactivated Tgfbr2fl alleles in cells that originate from the inner layer of the optic cup (OC), i.e., retinal neurons and Müller cells. Cre-negative littermates carrying floxed *Tgfbr2* alleles (*Tgfbr2^fl/fl^*) still express TGFBR2.

To genetically induce photoreceptor degeneration, the mice were additionally crossbred with hemizygous VPP mice carrying a rhodopsin mutant with point mutations at positions V20G, P27L, and P23H, in addition to wildtype rhodopsin [10]. The VPP mutation results in a progressive retinal neurodegeneration [10]. The resulting offspring analyzed in this study were as follows: Control mice (expressing wildtype rhodopsin and carrying *Tgfbr2^fl/fl^* alleles); *Tgfbr2^ΔOC^* mice (expressing wildtype rhodopsin and harboring a Cre-mediated deletion of *Tgfbr2* in retinal neurons and Müller cells); *Tgfbr2^fl/fl^*;VPP mice (henceforth termed ‘VPP mice’; expressing the VPP rhodopsin mutant protein) and *Tgfbr2^ΔOC^*;VPP mice (henceforth termed ‘double mutant mice’; expressing the VPP transgene in combination with Cre-mediated deletion of *Tgfbr2* in retinal neurons and Müller cells). All experiments were performed on mice of both sexes.

### 5.2. Genotyping and Tgfbr2 Deletion

Genotypes were screened by isolating genomic DNA from ear biopsies and tested by PCR analyses as previously described [9,12]. Briefly, for VPP genotyping, the following primers were used: 5′-agactgacatggggaggaattcccaga-3′ (sense) and 5′-gagctgctcgaagtgactccgacc-3′ (antisense). The thermal cycle protocol was denaturation at 94 °C for 30 s, annealing at 68 °C for 45 s and elongation at 72 °C for 45 s for 35 cycles. For Tgfbr2 genotyping we used the sense primer 5′-gcaggcatcaggacccagtttgatcc-3′ and the antisense primer 5′-agagtgaagccgtggtaggtgagcttg-3′ with the following thermal cycle protocol: denaturation at 95 °C for 30 s, annealing at 61 °C for 30 s and elongation at 72 °C for 45 s for 34 cycles. To genotype for the presence of the Cre recombinase we used the sense primer 5′-atgcttctgtccgtttgccg-3′ and the antisense primer 5′-cctgttttgcacgttcaccg-3′ with the thermal cycle protocol denaturation at 95 °C for 30 s, annealing at 60 °C for 30 s and elongation at 72 °C for 30 s for 34 cycles.

### 5.3. BaseScope^®^/In Situ Hybridization

In situ hybridization was performed as previously described [9]. Briefly, the eyes of one-month-old animals were enucleated and fixed for 4 h in 4% PFA. After washing in phosphate buffer (PB, 0.1 M, pH 7.4), the eyes were embedded in paraffin according to standard protocols. For in situ hybridization (ACD, Newark, NJ, USA), 6 mm thick paraffin sections were pre-treated with retrieval reagent and protease according to the user manual. BaseScope^TM^ Detection Reagent Kit v2—RED was used to label *TGF-β receptor type 2* (*Tgfbr2*) (ACD catalog number: 845871). The sections were analyzed on an Axio Imager Z1 microscope with the Apotome.2 function (Carl Zeiss, Jena, Germany) using Zeiss Zen software (Carl Zeiss, Jena, Germany).

### 5.4. RNA Sequencing

To perform RNA sequencing, total RNA of pooled retinae (right and left eye) of three-month-old mice was purified using the RNeasy Mini Kit by Qiagen (Venlo, The Netherlands). Library preparation and RNAseq were performed at the service facility ‘KFB—Center of Excellence for Fluorescent Bioanalytics’ (Regensburg, Germany. www.kfb-regensburg.de, accessed on 1 December 2021). Library preparation and RNAseq were carried out as described in the Illumina TruSeq Stranded mRNA Sample Preparation Guide, the Illumina NextSeq 500 System Guide (Illumina, Inc., San Diego, CA, USA), and the KAPA Library Quantification Kit—Illumina/ABI Prism User Guide (Kapa Biosystems, Inc., Woburn, MA, USA). In brief, 250 ng of total RNA was used for purifying the poly-A-containing mRNA molecules using poly-T oligo-attached magnetic beads. Following purification, the mRNA was fragmented to an average insert size of 200–400 bases using divalent cations under elevated temperature (94 °C for 4 min). Next, the cleaved RNA fragments were reverse transcribed into first strand cDNA using reverse transcriptase and random hexamer primers. Actinomycin D was added to improve strand specificity by preventing spurious DNA-dependent synthesis. Blunt-ended second strand cDNA was synthesized using DNA Polymerase I, RNase H, and dUTP nucleotides. The incorporation of dUTP, in place of dTTP, quenched the second strand during the later PCR amplification, because the polymerase does not incorporate past this nucleotide. The resulting cDNA fragments were adenylated at the 3′ ends; the indexing adapters were ligated; and, subsequently, specific cDNA libraries were created by PCR enrichment. The libraries were quantified using the KAPA SYBR FAST ABI Prism Library Quantification Kit. Equimolar amounts of each library were sequenced on a NextSeq 500 instrument controlled by the NextSeq Control Software (NCS) v2.2.0, using a 75 Cycles High Output Kit with the single index, paired-end (PE) run parameters. Image analysis and base calling were done with the Real Time Analysis Software (RTA) v2.4.11. The resulting. bcl files were converted into. fastq files with the CASAVA Software v1.8.2.

### 5.5. Bioinformatics

For all samples, at least 30 million reads were analyzed. Fastq files were quality controlled with FastQC v0.11.5. All files passed quality control. The reads were aligned against Ensembl Mus musculus GRCm38 version 94 using STAR aligner v2.5.3a. One sample (R21753) showed poor read alignments of less than 30% and was removed from further analyses. Reads were quantified using salmon v0.8.2. All subsequent analyses were conducted in R v3.5.1. Samples were screened for outliers using PCA and clustering analysis. One sample (R21741) was identified as an outlier and removed from further analyses. Thus, the final sample number was six control, five VPP, four control/VPP and four *Tgfbr2^ΔOC^*/VPP retinae. Transcriptional dysregulation was computed using tximport v1.10.0 and DESeq2 v1.22.1 with genotype as the variable of interest and sex of the mice as a covariate and using ashr as the fold change shrinkage estimator. The Benjamini–Hochberg procedure was used to correct for multiple comparisons (*p*-adjusted; *p_adj_*). For correlation network analysis, we used the normalized and variance stabilized counts from the DESeq2 analysis. Batch correction for sex was applied with limma v3.38.3, keeping the genotype as the variable of interest. The normalized, transformed, and batch corrected counts were used to construct a weighted gene correlation network using WGCNA v1.66. Heatmaps and k-mer analysis were carried out using ComplexHeatmap v2.3.2. Visualization was carried out using cytoscape v3.7.2 (http://cytoscape.org, accessed on 1 December 2021) with the Reactome FI app v7.2.1. Ontology analysis was carried out using the Enrichr website (https://maayanlab.cloud/Enrichr/, accessed on 1 December 2021). The scripts are available upon request.

### 5.6. Cell Death Measurement by TdT-Mediated dUTP-Biotin Nick End Labeling (TUNEL)

The number of degenerating photoreceptor cells was determined using TUNEL (DeadEnd Fluorometric TUNEL, Promega, Madison, WI, USA). This method was performed on retinae of one-month-old animals, as described previously [49,50]. TUNEL labeling was conducted on 4% paraformaldehyde-(PFA, in 0.1 M phosphate buffer (PP), pH 7.4) fixed and paraffin embedded eyes according to previously published protocols [51,52]. Labelled sections were visualized by fluorescence microscopy using the Axio Imager Z1 (Carl Zeiss, Jena, Germany). The total number of TUNEL-positive cells was normalized to the area of the ONL [mm^2^].

### 5.7. Light Microscopy and Spider Diagram Analyses

Eyes of three-month-old mice were carefully enucleated and fixed for 24 h in Ito’s fixative [53]. The eyes were marked with a thin, short metal needle at the superior limbus and embedded in Epon (Serva, Heidelberg, Germany). Semithin meridional sections (in nasal-temporal orientation) of 1.0 μm thickness were cut stretching through the optic nerve head (ONH) and the pupil. Sections were stained according to the Richardson’s protocol [54] and images taken using an Axio Imager Z1 light/fluorescent microscope (Carl Zeiss, Jena, Germany). The thickness of the outer nuclear layer (ONL) was measured at nine equidistant loci along the circumference of each hemisphere as described in [12,51,52,55]. The means and corresponding standard errors of the mean (SEM) were calculated for each measure point and the results were plotted as spider diagram.

### 5.8. Statistics

Data are expressed as mean ± SEM. Statistical comparative analyses between the mean variables of two individual test populations were performed using a two-tailed Student’s *t*-test in Excel (Microsoft Corporation, Redmond, WA, USA). One-way ANOVA analyses were performed in SPSS (IBM Corporation, Armonk, NY, USA) if more than two individual groups were compared (post-hoc test: Bonferroni). *p* values ≤ 0.05 were considered to be statistically significant.

## Figures and Tables

**Table 1:** Gene ontology and pathway enrichment analysis of genes that were only dysregulated in the double mutant mice.

Dysregulation Analysis	Enriched Pathways 1: WikiPathways 2019 Mouse; 2: KEGG 2019 Mouse; 3: BioPlanet 2019	Gene Ontology Enrichment (Biological Process 2021)	Potential Regulators 1: ChEA; 2: Encode TF ChIP-Seq 2015
310 genes significantly downregulated in double mutant, not regulated in VPP	1: Electron Transport Chain 20.83, Oxidative phosphorylation 18.40, Translation Factors 12.91, Proteasome Degradation 12.00, Cytoplasmic Ribosomal Proteins 8.45 2: Oxidative phosphorylation 18.90, Ribosome 16.84, RNA polymerase 13.09, Ubiquitin mediated proteolysis 11.34, Thermogenesis 8.26 3: Chemiosmotic coupling formation of ATP 134.04, Valine, leucine and isoleucine biosynthesis 62.79, Cytoplasmic ribosomal proteins 29.21, Cap-dependent translation initiation 24.60, Activation of mRNA upon binding of the cap-binding complex and eIFs, and subsequent binding to 43S 21.53	tRNA pseudouridine synthesis 181.21, regulation of stem cell division 107.93, sarcomere organization 75.56, phosphorylated carbohydrate dephosphorylation 62.79, inositol phosphate dephosphorylation 62.79	1: HCFC1 19.91, JARID1A 18.65, YY1 10.50, BCL3 7.26 2: KAT2A 43.14, EP300 19.63, SIN3A 19.63, POLR2AphosphoS5 17.07, MYC 16.31
337 genes significantly upregulated in double mutant, not regulated in VPP	1: Serotonin and anxiety 24.32, Dysregulated miRNA Targeting in Insulin/PI3K-AKT Signaling 12.95, IL-6 signaling Pathway 11.43, Oxidative Stress 11.37, Matrix Metalloproteinases 10.69, 2: Dopaminergic synapse 32.52, cAMP signaling pathway 17.02, IL-17 signaling pathway 13.61, Circadian entrainment 11.43, Aldosterone synthesis and secretion 10.74, 3: Activation of the AP-1 family of transcription factors 65.31, Erythropoietin-mediated neuroprotection through NF-kB 55.58, Signaling by Robo receptor 52.51, Inactivation of APC/C via direct inhibition of the APC/C complex 51.87, Kinesins 33.65	sinoatrial node cell differentiation 230.87, microtubule nucleation by microtubule organizing center 230.87, negative regulation of synapse organization 230.87, aromatic amino acid transport 161.57, snoRNA localization 161.57	1: SUZ12 30.34, JARID2 20.32, MTF2 17.81, EZH2 15.66, RING1B 15.25, 2: POLR2A 9.20

Enriched pathways and potential upstream regulators were predicted using the indicated databases. For gene ontology enrichment, only the top five non-redundant significantly enriched biological process terms are shown. The numbers following the terms are the combined score as calculated by Enrichr. Only terms with a combined score > 5 were considered.

**Table 2:** Gene ontology and pathway enrichment analysis of genes that were only dysregulated in the VPP mice.

Dysregulation Analysis	Enriched Pathways 1: WikiPathways 2019 Mouse; 2: KEGG 2019 Mouse; 3: BioPlanet 2019	Gene Ontology Enrichment (Biological Process 2021)	Potential Regulators 1: ChEA; 2: Encode TF ChIP-Seq 2015
1127 genes significantly downregulated in VPP, not regulated in double mutant	1: mRNA processing 15.87, Mismatch repair 11.66, Fatty Acid Biosynthesis 5.53, Eukaryotic Transcription Initiation 5.25 2: Basal transcription factors 23.11, RNA transport 18.65, Nucleotide excision repair 15.19, Mismatch repair 12.78, Lysine degradation 10.73 3: Small interfering RNA (siRNA) biogenesis 96.77, Cytoskeletal remodeling regulation and cell spreading by IPP complex components 48.54, RNA polymerase II C-terminal domain phosphorylation and interaction with capping enzyme 48.54, ATM-mediated phosphorylation of repair proteins 39.86, NOSTRIN-mediated endothelial NOS trafficking 39.86	mRNA cleavage involved in gene silencing by miRNA 161.54, cellular lipid biosynthetic process 96.77, snRNA modification 96.77, transcription-dependent tethering of RNA polymerase II gene DNA at nuclear periphery 96.77, mRNA splice site selection 76.01	1: KDM5B 36.25, CREM 24.91, FOXO3 20.56, BCL3 18.68 ERG 17.39 2: GABPA 59.59, KAT2A 49.90, MAX 44.05, FLI1 32.08, HCFC1 27.13
979 genes significantly upregulated in VPP, not regulated in double mutants	1: Glutathione metabolism 20.55, Fatty Acid Biosynthesis 16.81, Prostaglandin Synthesis and Regulation 15.42, ACE Inhibitor Pathway 14.90, Heme Biosynthesis 14.90 2: Folate biosynthesis 38.75, Propanoate metabolism 15.42, Oxidative phosphorylation 14.95, beta-Alanine metabolism 14.38, Nitrogen metabolism 12.71 3: Bile salt and organic anion SLC transporters 60.31, Catalytic cycle of mammalian FMOs 49.72, Kit receptor transcriptional targets 49.72, Second messenger role in netrin-1 signaling 37.78, Tetrahydrobiopterin (BH4) biosynthesis, recycling, salvage and regulation 37.78, Cell cycle negative regulation by p75 neurotrophin receptor 33.66	negative regulation of T cell migration 199.52, blood vessel endothelial cell proliferation involved in sprouting angiogenesis 120.22, basement membrane assembly 116.02, dolichyl diphosphate biosynthetic process 82.52, tetrahydrobiopterin metabolic process 73.01	1: SUZ12 12.02, THRA 10.23, SOX9 8.81, SRY 6.88, MTF2 5.96 2: n.s.

Enriched pathways and potential upstream regulators were predicted using the indicated databases. For gene ontology enrichment, only the top five non-redundant significantly enriched biological process terms are shown. The numbers following the terms are the combined score as calculated by Enrichr. Only terms with a combined score > 5 were considered. n.s. = no significant enrichment.

## Data Availability

The raw data files of the RNAseq data (Supplementary Tables S1–S3) are immediately available from the authors upon request and will later on be deposited in the NCBI GEO database under the current manuscript title.

## Supplementary Materials

The following supporting information can be downloaded at: https://www.mdpi.com/article/10.3390/ijms23052626/s1.

## Abbreviations

AAV	Adeno-associated virus
AMD	Age related macular degeneration
AP1	Activator protein 1
Bdnf	Brain derived neuroprotective factor
Cd29	Integrin beta1
CNS	Central nervous system
Cralbp	Cellular retinaldehyde-binding protein
Erk	Extracellular-signal regulated kinase
Fos	Fos proto-oncogene
GCL	Ganglion cell layer
Glul	Glutamine synthetase
Hddc	HD Domain containing 3
Il6	Interleukin-6
INL	Inner nuclear layer
Itgb1	Integrin beta-1
Jnk3	c-Jun N-terminal kinase3
MAP	Mitogen-activated protein
MAPK	Mitogen-activated protein kinase
Mesh1	HD Domain containing 3
Mrpl48ps	Mitochondrial ribosomal protein L48 pseudogene
Myo7a	Myosin VIIA
Ngf	Neurotrophins nerve growth factor
OC	Optic nerve
ONH	Optic nerve head
ONL	Outer nuclear layer
OPL	Outer plexiform layer
OS	Ora serrata
PB	Phosphate buffer
PFA	Paraformaldehyde
pr. co.	Probe control
Rhox4c	Reproductive homeobox 4C
Rlbp1	Retinaldehyde-binding protein 1
RNAseq	Next generation RNA sequencing
RPE	Retinal pigment epithelium
Smad	Mothers against decapentaplegic homolog
Tgf	Transforming growth factor
Tgfbr2	Transforming growth factor receptor
Tnf	Tumor necrosis factor
Trem2	Triggering receptor expressed on Myeloid cells 2
WGCNA	Weighted gene correlation network analysis
