# Supplementary figures and images for: Deficiency in Retinal TGFβ Signaling Aggravates Neurodegeneration by Modulating Pro-Apoptotic and MAP Kinase Pathways
Source: Int J Mol Sci. 2022 Feb 27;23(5):2626. doi: 10.3390/ijms23052626 (PMC8910086; doi:10.3390/ijms23052626)

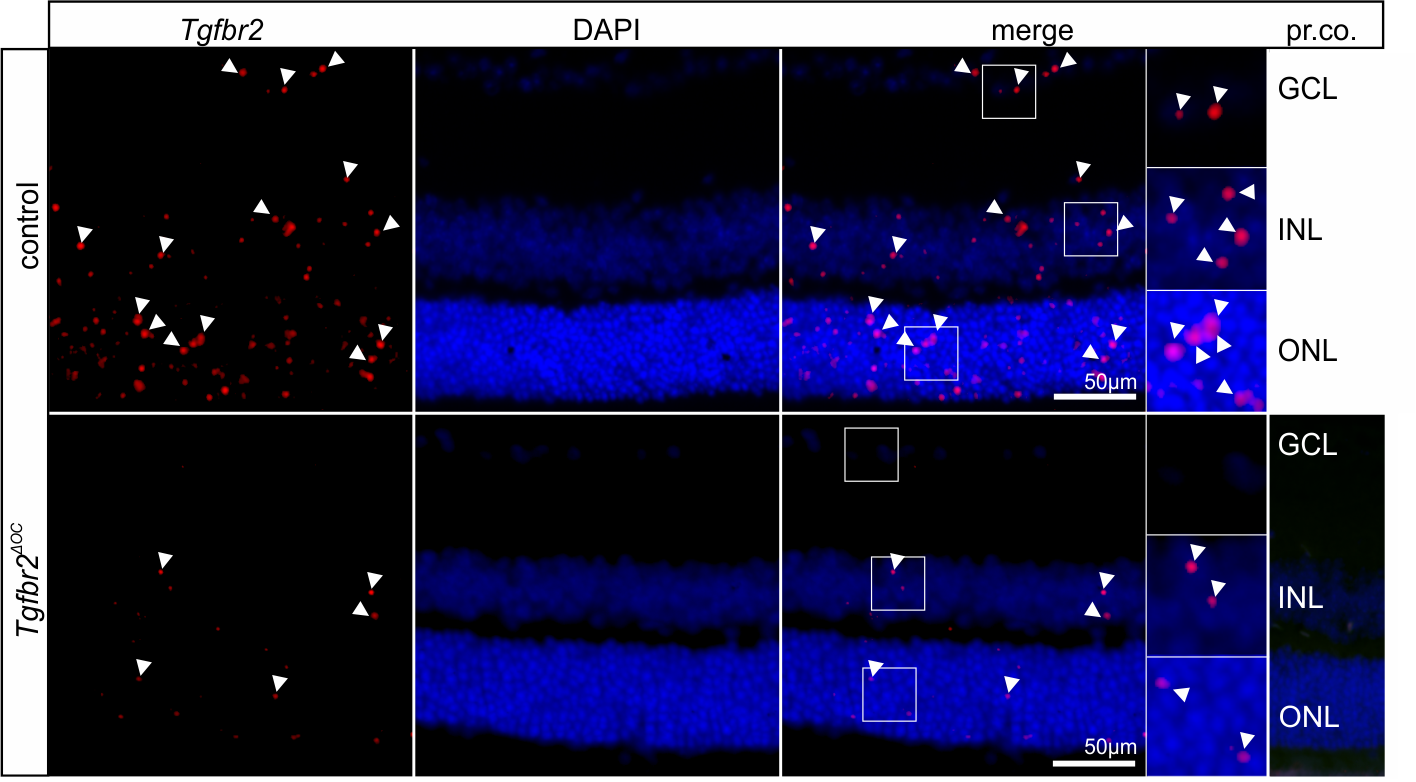

Supplement: Supplementary file 1 [file ijms-23-02626-s001.zip › suppl_Fig S1_Tgfbr2 BaseScope.tif]

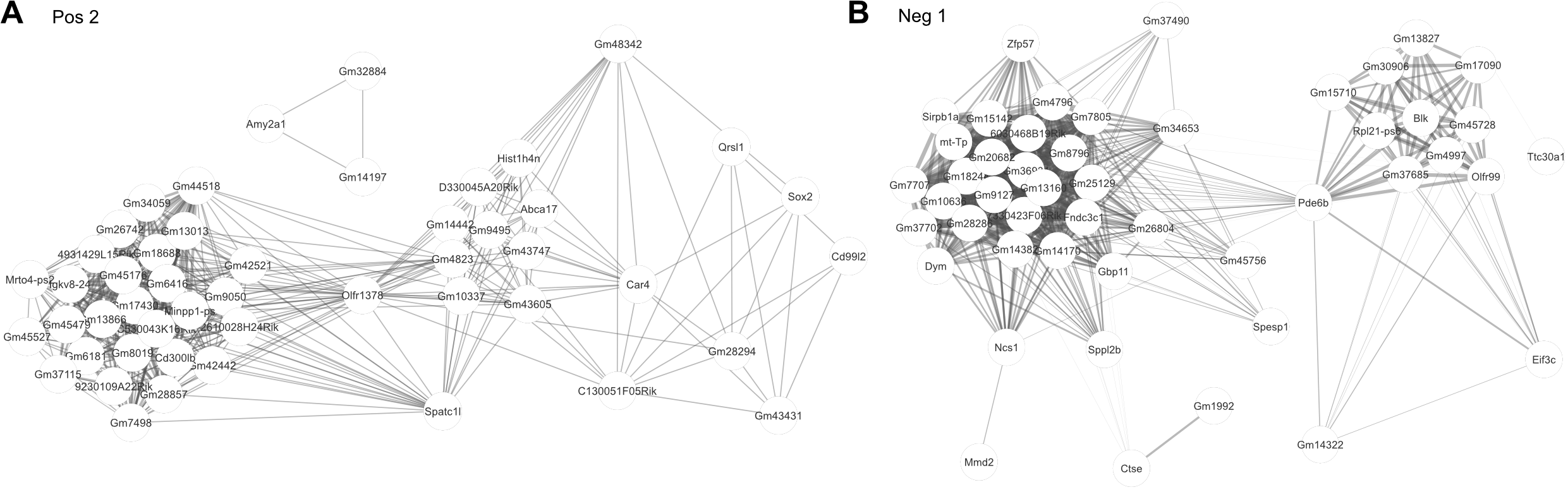

Supplement: Supplementary file 1 [file ijms-23-02626-s001.zip › suppl_Fig S2.tif]

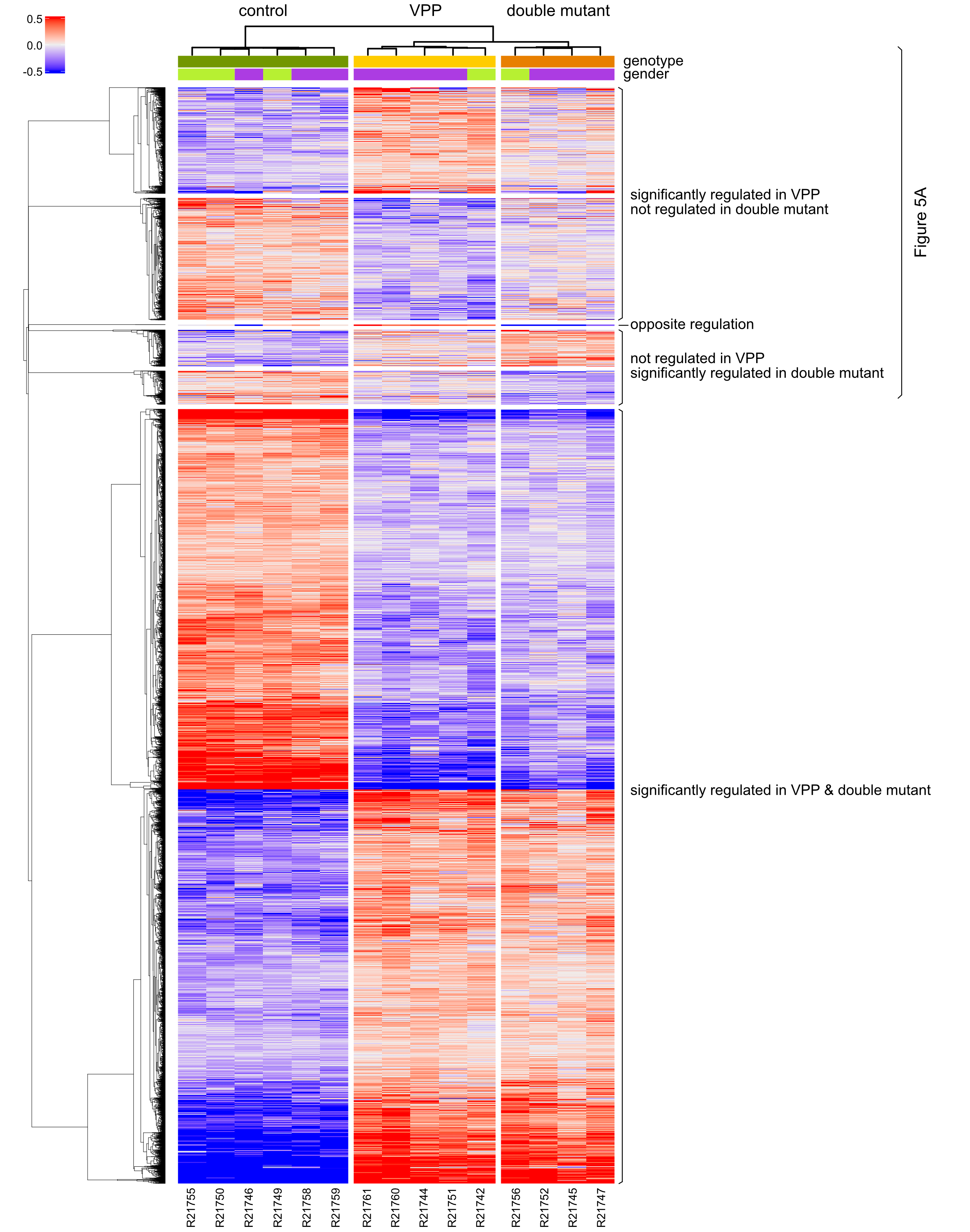

Supplement: Supplementary file 1 [file ijms-23-02626-s001.zip › suppl_Fig S3.tif]
